# Barriers to prevent second-hand smoke (SHS) exposure among pregnant women and children in Egypt

**DOI:** 10.1093/eurpub/ckac131.438

**Published:** 2022-10-25

**Authors:** Z Hassanein, T Langley, I Bogdanovica, R Murray

**Affiliations:** 1 Academic Unit Lifespan and Population Health, University of Nottingham, Nottingham, UK; 2 Public Health Department, Assiut University, Assiut, Egypt

## Abstract

**Background:**

The prevalence of daily second-hand smoke (SHS) exposure among pregnant non-smoking women and children in Egypt is estimated to be about 50% and 55%, respectively. This study aimed to explore barriers to preventing SHS exposure among pregnant women/children and smoking behavior at home in Egypt.

**Methods:**

Focus group discussions (FGDs) with pregnant women/mothers of children residing in urban/rural areas (n = 61). Data were coded and analyzed thematically.

**Results:**

61 participants were recruited, aged 18-49. They reported being never smokers and SHS exposure for themselves and their children was mainly at home. Pregnant women/mothers had some general knowledge of the dangers of SHS, but their knowledge appeared incomplete. The most commonly reported barriers to preventing SHS exposure/adopting a smoke free home or workplace were having men who smoke in the household, doctors not being supportive regarding smoking cessation, SHS exposure is socially accepted and fear among women of damaging a relationship; being nervous about asking smokers to stop, and being worried about disputes and arguments with husband. The majority of interviewees’ families were reported to allow smoking anywhere in the home; others implemented some measures to prevent SHS, however, these tended to be inconsistently implemented and unlikely to be effective.

**Conclusions:**

This study increases our knowledge of the barriers of non-smoking Egyptian pregnant women/mother of children in creating and maintaining smoke free environment for themselves and their children. There is a need to denormalise SHS exposure and better enforcement of smoke free policies.

**Key messages:**

• Better enforcement of smoke free policies, and more support for smoking cession services are needed in Egypt.

• SHS policy, practice, and research should focus on male family members to increase their effectiveness.

